# Georgia Medical Association

**Published:** 1873-01

**Authors:** 


GEORGIA MEDICAL ASSOCIATION.—We desire to remind the profession of the State that this body holds its next annual meeting (April, 1873,) in the city of Atlanta. The Secretary will doubtless arrange with the railroads for the usual reduction of fare, and the profession here will cordially welcome every medical gentleman in the State. We are confident that the approaching session will exceed in numbers, ability and contributions to science, any preceding meeting of the body, and that it will more firmly cement the fraternal feeling so happily inaugurated at the meeting last year in Columbus. We are sure that no Southern State contains more medical talent than is found in the State of Georgia, and it is earnestly urged upon the profession throughout its entire borders to come up in strong force, and exhibit, through our transactions of the present year, a fair index of the real ability of the Georgia medical profession.

